# Integrating pathway elucidation with yeast engineering to produce polpunonic acid the precursor of the anti-obesity agent celastrol

**DOI:** 10.1186/s12934-020-1284-9

**Published:** 2020-01-28

**Authors:** Nikolaj L. Hansen, Karel Miettinen, Yong Zhao, Codruta Ignea, Aggeliki Andreadelli, Morten H. Raadam, Antonios M. Makris, Birger L. Møller, Dan Stærk, Søren Bak, Sotirios C. Kampranis

**Affiliations:** 1 Plant Biochemistry Section, Department of Plant and Environmental Sciences, University of Copenhagen, Thorvaldsensvej 40, 1871 Frederiksberg C, Denmark; 2 Department of Drug Design and Pharmacology, Faculty of Health and Medical Sciences, University of Copenhagen, Universitetsparken 2, 2100 Copenhagen, Denmark; 3 Institute of Applied Biosciences-Centre for Research and Technology Hellas (INAB-CERTH), P.O. Box 60361, 57001 Thermi, Thessaloniki Greece

**Keywords:** P450, Terpenoid, Tobacco, Biosynthetic pathway

## Abstract

**Background:**

Celastrol is a promising anti-obesity agent that acts as a sensitizer of the protein hormone leptin. Despite its potent activity, a sustainable source of celastrol and celastrol derivatives for further pharmacological studies is lacking.

**Results:**

To elucidate the celastrol biosynthetic pathway and reconstruct it in *Saccharomyces cerevisiae*, we mined a root-transcriptome of *Tripterygium wilfordii* and identified four oxidosqualene cyclases and 49 cytochrome P450s as candidates to be involved in the early steps of celastrol biosynthesis. Using functional screening of the candidate genes in *Nicotiana benthamiana*, *Tw*OSC4 was characterized as a novel oxidosqualene cyclase that produces friedelin, the presumed triterpenoid backbone of celastrol. In addition, three P450s (CYP712K1, CYP712K2, and CYP712K3) that act downstream of *Tw*OSC4 were found to effectively oxidize friedelin and form the likely celastrol biosynthesis intermediates 29-hydroxy-friedelin and polpunonic acid. To facilitate production of friedelin, the yeast strain AM254 was constructed by deleting *UBC7*, which afforded a fivefold increase in friedelin titer. This platform was further expanded with CYP712K1 to produce polpunonic acid and a method for the facile extraction of products from the yeast culture medium, resulting in polpunonic acid titers of 1.4 mg/L.

**Conclusion:**

Our study elucidates the early steps of celastrol biosynthesis and paves the way for future biotechnological production of this pharmacologically promising compound in engineered yeast strains.

## Background

Obesity is a major health risk associated with numerous debilitating conditions, including type 2 diabetes, cardiovascular disease and hypertension [1]. Despite intense research, an effective pharmacological treatment of obesity is still lacking. One promising approach involves leptin, an adipocyte-derived protein hormone that sends signals that inform the central nervous system on the status of the peripheral energy stores, controlling overall metabolism and food uptake [2, 3]. In obesity, cells become unresponsive to leptin [4–6]. Thus, identifying molecules that alleviate leptin resistance could provide a treatment for the condition. In a large search for leptin sensitizers, the plant specialized metabolite celastrol was identified as the most potent candidate, leading to up to 45% weight loss in hyperleptinemic diet-induced obese mice [7]. Celastrol was shown to protect against obesity and metabolic dysfunction through activation of the transcription factor HSF1, which regulates metabolic programs in adipose tissue and muscle [8]. Further studies showed that celastrol binds specifically to the nuclear receptor Nur77, which may confer the leptin-sensitizing effect by regulating HFD-induced hypothalamic inflammation [9]. In addition, celastrol has been found to have a strong protective effect in several other human conditions [10–19].

Celastrol is a red/orange quinone methide nor-triterpenoid found in the root bark of some genera of *Celastraceae*, including the traditional Chinese medicinal plant *Tripterygium wilfordii* [20, 21]. However, scalable and reliable sourcing of celastrol or related compounds from plant roots is challenged by slow growth, yield fluctuations and complexity of harvesting branching roots. Furthermore, the production is geographically restricted as the export of *T. wilfordii* from its natural habitat is regulated by the Nagoya protocol. Although total organic synthesis of celastrol has been reported, it is laborious and inefficient [22]. Thus, sourcing from nature or chemical synthesis does not provide sufficient amounts of pure compound. Therefore, alternative celastrol production methods are needed.

Heterologous reconstruction of biosynthetic pathways in engineered microorganisms is an attractive solution for the sustainable production of structurally complex specialized metabolites. In the case of celastrol, this approach will enable its efficient stereo-specific biosynthesis and the production of derivatives in adequate amounts. The model organism *Saccharomyces cerevisiae* (baker’s yeast) is an established host for the efficient production of industrial chemicals and complex high-value compounds [23–25]. This is due to its amenability to genetic engineering, relative absence of secondary metabolites, and its compatibility with most key eukaryotic biosynthetic enzymes, such as cytochromes P450 (P450s), enabling the reconstruction of complete biosynthetic pathways.

The ability to produce high-value compounds in engineered microorganisms requires elucidation of the biosynthetic pathway of the compound of interest. To facilitate pathway elucidation, transient gene expression in tobacco (*Nicotiana benthamiana*) leaves is an efficient tool for in vivo characterization of novel plant biosynthetic enzymes [26–29]. Compared with microbial systems, such as yeast, transient tobacco expression offers a plant molecular environment that may be functionally important for plant-derived enzymes. For example, plants differ from yeast in the lipid composition of the endoplasmic reticulum (ER) membrane [30, 31], which may influence the correct insertion or function of membrane-associated proteins, such as OSCs or P450s. Membrane composition may also affect the transport of hydrophobic specialized metabolites, like terpenes, between different cellular compartments (i.e., chloroplasts and ER), or between membrane-bound enzymes catalyzing successive steps of a pathway. In addition, plants express redox co-factors dedicated to secondary metabolism, such as specialized cytochrome b5 proteins [32], and many CYPs depend on such co-factors for optimal activity.

**Fig. 1:**
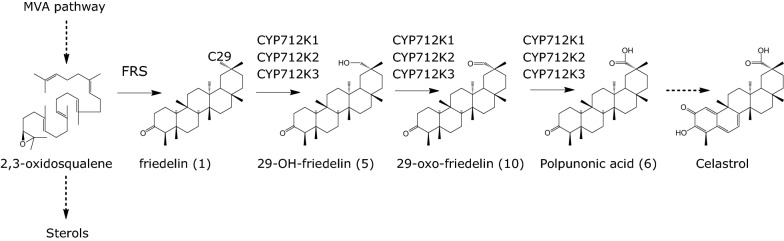
Proposed celastrol biosynthetic pathway. Dashed arrows depict multiple enzymatic steps. Friedelin synthase (FRS) performs the first committed step of the pathway by cyclization of the common triterpenoid precursor 2,3-oxidosqualene into friedelin. Next, CYP712K1, CYP712K2 or CYP712K3 catalyzes successive oxygenations of friedelin to form polpunonic acid (**6**) via 29-hydroxy-friedelin (**5**) and 29-oxo-friedelin (**10**). Enzymes of different types are proposed to perform the rest of the reactions to obtain celastrol

Celastrol biosynthesis likely adheres to the common triterpenoid scheme that starts with cycloisomerization of 2,3-oxidosqualene by an oxidosqualene cyclase (OSC) and continues with oxidative decoration(s) of the 30-carbon triterpenoid core structure by cytochrome P450 enzymes (Fig. 1) [33, 34]. Downstream of these initial oxygenation steps, other enzyme families such as acyl transferases, dehydrogenases, and 2-oxoglutarate-dependent dioxygenases modify plant terpenoids [27, 35, 36]. In the case of celastrol biosynthesis, the core triterpenoid scaffold is thought to be friedelin (**1**), synthesized by OSC enzymes named friedelin synthases (FRSs) [37, 38]. However, knowledge of the downstream biosynthetic steps is lacking, hindering biotechnological production of celastrol in a cell factory.

Here, we address this challenge by combining pathway elucidation with metabolic engineering to identify missing enzyme activities and establish production of celastrol precursors in an engineered yeast cell factory. We describe the establishment of a platform for the bio-production of celastrol and related celastroids in yeast through initial enzyme activity screening in tobacco leaves, followed by structural characterization of the produced compounds and construction of efficient yeast production strains that allows for optimization of yeast growth and product extraction. These findings provide a base for future biotechnological production of pharmacologically relevant anti-obesity celastroids.

## Results

### Transcriptome mining identified friedelin synthase candidates

**Fig. 2:**
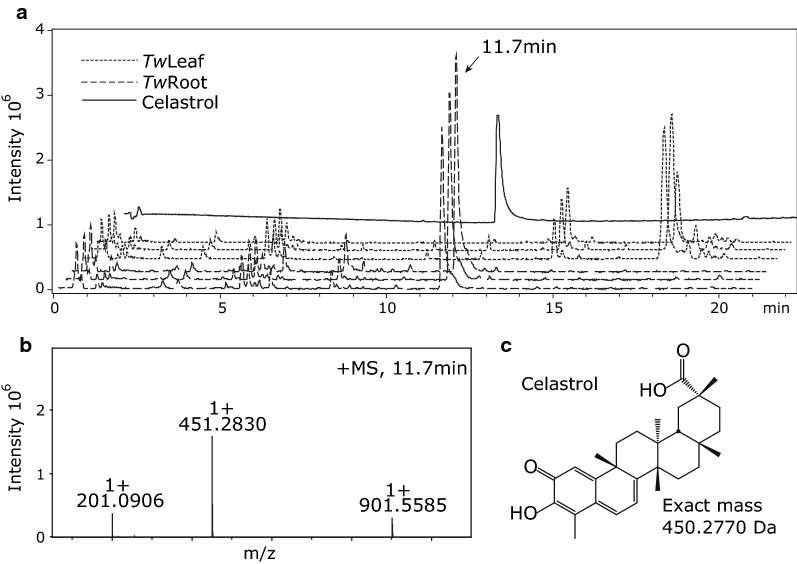
LC–ESI–MS profiles of methanol extracts of *Tripterygium wilfordii* root and leaves. **a** Methanol extracts of root tissue (three biological replicates; dashed line) show a strong peak at 11.7 min with m/z of 451.2830 [M+H]^+^ corresponding to celastrol. The identity of the compound was confirmed using a celastrol standard (solid line). **b** Mass spectrum of celastrol eluting at 11.7 min. **c** Structure of celastrol

Establishing microbial production of celastrol and its precursors requires elucidation of the biosynthetic pathway and identification of the enzymes involved. To facilitate this, we based our search for candidate biosynthetic genes on transcriptomic data [39] derived from root tissue of *T. wilfordii* confirmed to produce high levels of celastrol (Fig. 2).

The canonical biosynthesis of triterpenoids begins with carbon-backbone formation via cyclization of 2,3-oxidosqualene by an OSC. The dataset was mined for OSC transcripts with the MassBlast transcriptome analysis tool [40], using the *Arabidopsis thaliana* β-amyrin synthase [41] (*At*BAS; accession: BAG82628) as query sequence. Candidate OSCs were selected on the basis of amino acid sequence similarity with *At*BAS (a representative OSC of plant specialized metabolism) and prioritized by relative transcript abundance (Fragments Per Kilobase Million, FPKM). We identified a set of four candidate OSCs and named them *Tw*OSC1 (19 FPKM), *Tw*OSC2 (1 FPKM), *Tw*OSC3 (28 FPKM), and *Tw*OSC4 (245 FPKM), reflecting the order of their similarity with the query sequence. Indication of the expression levels of the four genes assessed by relative transcript numbers (FPKM) highlighted *Tw*OSC4 as the most abundant OSC transcript.

### *Tw*OSC4 synthesizes friedelin in tobacco

**Fig. 3:**
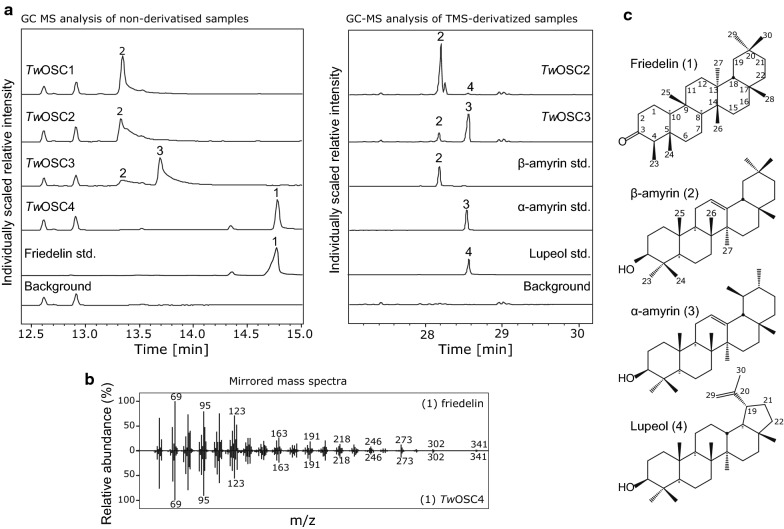
Characterization of *T. wilfordii* candidate oxidosqualene cyclases by transient expression in tobacco. **a** Two discs (Ø3 cm) of leaves expressing the indicated enzymes were extracted in 1 mL hexane (left panel) or 1 mL ethyl acetate (right panel) and analyzed by GC–MS either directly (left panel) or after solvent evaporation (of 0.15 mL aliquots) and trimethylsilyl cyanide derivatization (right panel). Major products were established by comparison of mass spectra and retention time to authentic standards (see also Additional file 1: Figure S1). **b** Comparison of the mass spectrum of friedelin standard with the spectrum of the main product of *Tw*OSC4. **c** Structures and selected carbon numbering of the products

To allow for characterization of the candidate enzymes in a host well-suited for plant enzymes, we chose to express the OSC candidates by *Agrobacterium*-mediated transient expression in tobacco. Tobacco cells naturally provide endogenous 2,3-oxidosqualene, the archetypical substrate of OSCs, and the physiologically-relevant cellular environment to handle the highly hydrophobic OSC products. The full-length coding sequences of the four candidate synthases were amplified from *T. wilfordii* root cDNA by PCR and cloned into the *A. tumefaciens* binary vector pCAMBIA-1300-35Su [42]. Following transient expression of the individual candidate OSCs, we traced the apolar metabolite profile of tobacco leaves by GC–MS analysis of both hexane extracts or derivatized residues of dried ethyl acetate extracts (to cover both derivatizable and non-derivatizable compounds). When compared to an empty vector control, all four candidates yielded novel peaks in the GC–MS analysis (Fig. 3a). Transient expression of one of the candidates, *Tw*OSC4, resulted in a predominant peak that corresponded to friedelin, as identified by comparison of retention time and mass spectrum to an authentic standard (Fig. 3a, b). Only a minor additional new peak was observed in the chromatogram for *Tw*OSC4, which, however, also appeared in the friedelin standard, suggesting that *Tw*OSC4 is a highly product specific friedelin synthase. Transient expression of *Tw*OSC1 and *Tw*OSC2 resulted in new peaks with the same retention time and mass spectra as an authentic β-amyrin (**2**) standard (Fig. 3a and Additional file 1: Figure S1). The remaining synthase, *Tw*OSC3, produced mainly α-amyrin (**3**) together with relative lower amounts of β-amyrin (**2**), as confirmed by comparison to authentic standards. In addition, *Tw*OSC2 also produced minor amounts of lupeol (**4**).

### Transcriptome mining identified 49 candidate CYPs

Celastrol carries several oxidative decorations on its core structure (Fig. 2). To identify the enzymes catalyzing these oxygenations, we targeted the large family of plant P450s as they are commonly associated with carbon oxygenations of hydrophobic molecules. 49 candidate P450 genes were selected based on homology with known terpene-targeting P450s and by displaying relatively high FPKM values. Of these candidates, 42 P450 candidates were identified from the previously reported *T. wilfordii* root transcriptome [39]. The remaining seven candidates were selected from an earlier published leaf transcriptome of *T. wilfordii* [43]. The P450 candidates were assigned names by Professor David Nelson (University of Tennessee, USA) according to the P450 nomenclature system. P450 families typically associated with specialized metabolite biosynthesis were well represented in the list of candidates including, among others, nine members of the CYP71 family, 14 members the CYP88 family and nine members of the CYP82 family (P450 names listed in Additional file 1: Table S1).

### CYP712K1, CYP712K2 and CYP712K3 oxygenate friedelin

**Fig. 4:**
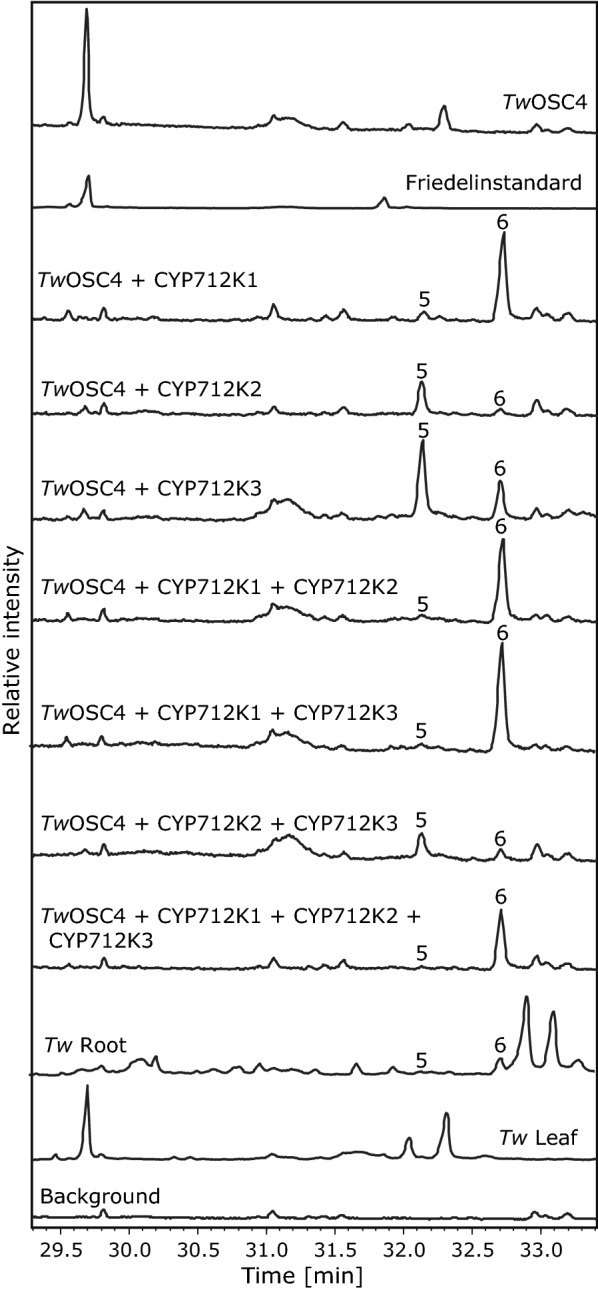
Functional characterization of CYP712K1, CYP712K2, and CYP712K3 using tobacco agroinfiltration and GC–MS analysis. The three P450s were analyzed for their ability to oxygenate friedelin, produced by co-infiltration with *TwOSC4*. Metabolites of leaves co-expressing indicated enzymes were detected by GC–MS analysis of dried and TMS derivatized leaf disc ethyl acetate extracts. Co-infiltration with *p19* and *CfDXS* was used in all experiments to inhibit silencing of foreign gene expression and to elevate the isoprenoid diphosphate substrate pool, respectively

The 49 selected P450 candidates were cloned into appropriate vectors for biochemical analysis using transient expression in tobacco leaves. Following agrobacterium mediated co-infiltration of tobacco leaves with candidate P450s and friedelin synthase (*Tw*OSC4), the ability of each P450 to oxygenate friedelin was evaluated by GC–MS analysis of TMS derivatized leaf-extracts. We identified three P450s of the CYP712 family (CYP712K1, CYP712K2 and CYP712K3), which, when co-expressed with *Tw*OSC4, caused the appearance of two novel constituents, corresponding to compounds **5** and **6**, in conjunction with significant reduction of friedelin. The levels and ratio of the novel constituents varied depending on the P450 enzyme (Fig. 4), with CYP712K1 favoring formation of **6** and CYP712K2 favoring formation of **5**. CYP712K3 also favored production of **5**, albeit to a lesser extent. The combined effect of the three P450s on the product profile was assessed by co-expressing the three possible two-P450 combinations and all three P450s together in the presence of *Tw*OSC4 (Fig. 4). These combinations revealed that CYP712K1 consistently determined the product ratio in favor of **6**, independently of the presence of the other two CYP712s. This finding was interpreted as either a consequence of CYP712K1 having greater affinity for the substrate, or simply **5** being an intermediate en route to **6**. Since no additional products were observed when any two of the three P450s were combined, we concluded that these enzymes must have the same regiospecific activities. To evaluate the substrate promiscuity of the isolated enzymes, the ability of CYP712K1 and CYP712K2 to oxygenate α- or β-amyrin by agrobacterium mediated co-infiltration with *Tw*OSC3- and *Tw*OSC2-were evaluated (Additional file 1: Figure S2). Both enzymes oxygenated β-amyrin to products **7** and **8**, which may be consecutive oxygenation products of the β-amyrin scaffold. CYP712K2, but not CYP712K1, was also able to oxygenate α-amyrin to compound **9**. Since these products are unlikely to be intermediates of the celastrol pathway, they were not studied further.

To investigate the existence of friedelin and compounds representing peaks **5** and **6** in roots and leaves of *T. wilfordii,* plant extracts of these tissues were also analyzed by GC–MS (Fig. 4). Both **5** and **6**, but not friedelin, were detected in the root extract. By contrast, compounds **5** and **6** were not observed in the leaves, which, on the other hand, accumulated friedelin. This is consistent with friedelin being specifically converted to celastrol and related compounds in the roots.

### Structural elucidation of the CYP712K1, CYP712K2 and CYP712K3-catalyzed products

**Fig. 5:**
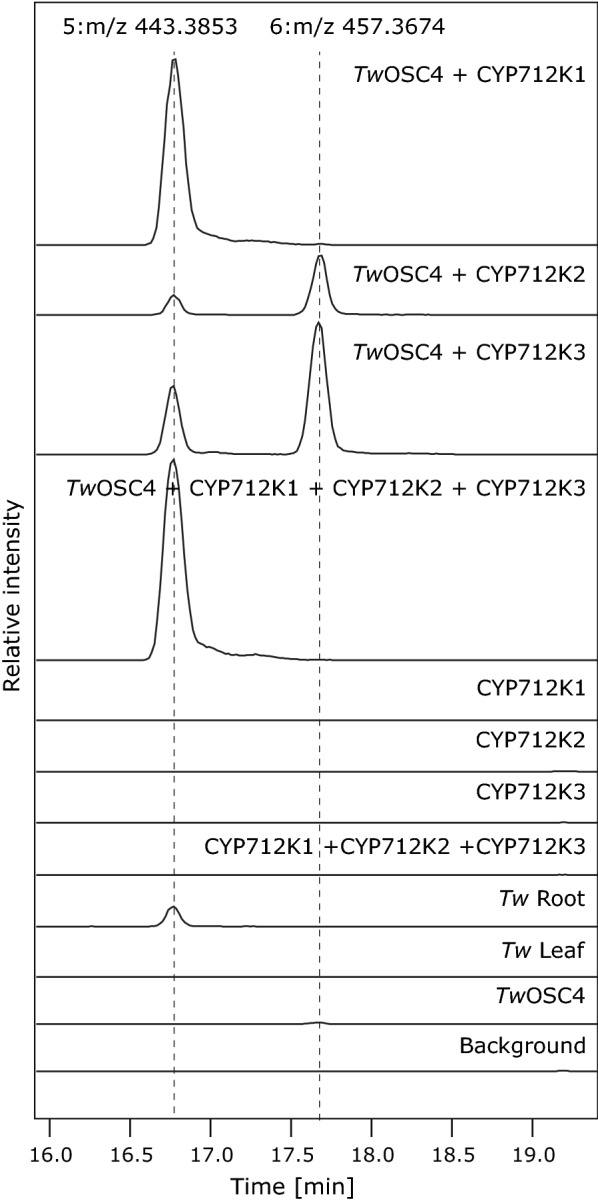
Transient expression of *T. wilfordii* CYP712K1, CYP712K2 and CYP712K3 in tobacco leaves for the determination of the exact masses of the products formed. Metabolites formed in in response to the co-expression of the indicated enzymes were detected by LC–ESI–MS analysis (showing EIC: m/z 443.35; 457.35 ± 0.05 [M+H]^+^) of leaf disc methanol extracts. Co-infiltration with *p19* and *CfDXS* was used to inhibit silencing of foreign gene expression and to elevate the isoprenoid diphosphate substrate pool, respectively

To confirm that the products formed by the action of the *Tw*OSCs and CYP712K1, CYP712K2 and CYP712K3 were indeed celastrol precursors, the structures of the novel compounds **5** and **6** were elucidated. First, exact mass determination was obtained by liquid chromatography-electrospray ionization mass spectrometry (LC–ESI–MS; Fig. 5). The accurate mass to charge ratio of was 443.3853 m/z [M+H]^+^ for compound **5** and 457.3674 m/z [M+H]^+^ for compound **6,** corresponding to an approximate m/z gain of 16 and 30, respectively, compared to friedelin (predicted m/z 427.3940 [M+H]^+^). These mass gains are consistent with the formation of **5** and **6** by a single and two oxygenations, respectively. Such oxygenations could correspond to a carbon hydroxylation (+ 16) and an additional oxygenation of a carbonyl into a carboxyl group (+ 30). These results suggest that these P450 enzymes likely catalyze three sequential oxygenations of friedelin into an alcohol, and further to a carboxylic acid via an aldehyde. This mechanism is consistent with the action of other diterpene and triterpene modifying P450s that catalyze three successive oxidation steps on the same carbon [44–46].

In order to unambiguously elucidate the structure of compounds **5** and **6** using NMR, we isolated these compounds from large-scale agro-infiltration experiments in tobacco (48 plants for each compound). To preferentially produce compound **5**, tobacco plants were co-infiltrated with *TwOSC4* and *CYP712K3*. To produce compound **6**, *TwOSC4* and *CYP712K1* were co-infiltrated. In both cases together with the *p19* suppressor of silencing and the gene encoding 1-deoxy-d-xylulose 5-phosphate synthase *CfDXS*. NMR analysis of the isolated compounds and comparison to previously reported data [47, 48] identified compound **5** as 29-hydroxy-friedelin and compound **6** as polpunonic acid (29-carboxy-friedelin; Fig. 1, Additional file 1: Table S3). Combined with our GC–MS and LC–ESI–MS results, and the combinatorial experiment in the section above describing the functional analysis of the different CYP712K enzymes, these findings confirm that all three enzymes catalyze the three-step oxygenation of C-29 of friedelin to form polpunonic acid through 29-hydroxyfriedelin and 29-oxofriedelin (Fig. 1).

### Friedelin production in yeast

Having identified biosynthetic enzymes able to produce polpunonic acid (**6**), we set out to reconstitute the pathway in *S. cerevisiae,* and to establish a platform for the heterologous production of various celastroids. To establish friedelin production in yeast, the gene encoding the *T. wilfordii* friedelin synthase *Tw*OSC4 was cloned into the yeast expression plasmid pUUS and introduced into the diploid yeast strain AM109, specifically designed for terpenoid production [49] (Table 1), resulting in strain AM109-2. Following galactose induction and growth, the yeast cells were disrupted by saponification, extracted with hexane and the organic extract analyzed by GC–MS. However, we were unable to detect production of friedelin despite being able to observe production of another related triterpene, α-amyrin, by the *Salvia pomifera* α-amyrin synthase (*Sp*AS1) [50] under the same conditions, suggesting that *Tw*OSC4 may not be active in this yeast system.

**Table 1:** Yeast strains used in this study

Strain	Description	Source
AM109	Mat a/α, P_*GAL1*_-HMG2(K6R):: HOX2, *ura3, trp1, his3*, P_*TDH3*_-HMG2(K6R)X2-::*leu2, ERG9/erg9, UBC7/ubc7, SSM4/ssm4, PHO86/pho86*	Ignea et al. [49]
AM109-1	AM109; pUUS(empty vector)	This study
AM109-2	AM109; pUUS(*Tw*OSC4)	This study
AM109-3	AM109; pUUS(*Kd*FRS)	This study
AM238	MATα *his3, ura3, trp1, rox1, dos2, yer134c, vba5, ynr063w, ygr259c*	Trikka et al. [50]
AM238-1	AM238; pUUS(empty vector)	This study
AM238-2	AM238; pUUS(*Kd*FRS)	This study
AM254	AM238; *ucb7*	This study
AM254-1	AM254; pUUS(empty vector)	This study
AM254-2	AM254; pUUS(*Kd*FRS)	This study
AM254-3	AM254; pUUS(empty vector); pHTDH(*Sc*tHMG2); pESC-LEU(empty vector), pESC-TRP(empty vector)	This study
AM254-4	AM254; pUUS(*Kd*FRS); pHTDH(*Sc*tHMG2); pESC-LEU(empty vector); pESC-TRP(empty vector)	This study
AM254-5	AM254; pUUS(*Kd*FRS); pHTDH(*Sc*tHMG2); pESC-LEU(*Tw*CPR1); pESC–TRP(CYP712K1)	This study
AM254-6	AM254; pUUS(*Kd*FRS); pHTDH(*Sc*tHMG2); pESC-LEU(*Tw*CPR1); pESC-TRP(CYP712K2)	This study
AM254-7	AM254; pUUS(*Kd*FRS); pHTDH(*Sc*tHMG2); pESC-LEU(*Tw*CPR1); pESC-TRP(CYP712K3)	This study

**Fig. 6:**
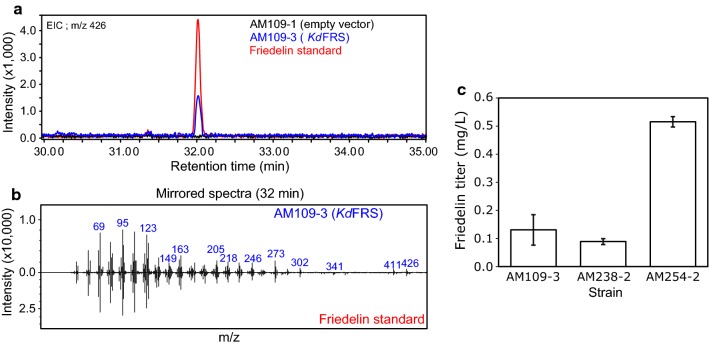
Functional analysis of *Kd*FRS in yeast. **a** Comparison of extracted ion GC–MS chromatograms at m/z 426 (friedelin parent mass) of hexane extracts of the strains AM109-3 (*Kd*FRS, blue), control strain AM109-1 (empty vector, black), and authentic friedelin standard (red), showing accumulation of friedelin in AM109-3. **b** The constituent eluting at 32 min retention time shows the same mass spectrum as the friedelin standard. **c** Friedelin accumulation in yeast strains engineered for terpenoid production. Total friedelin production in the strains AM109-3, AM238-2 and AM254-2 expressing *Kd*FRS was quantified by LC–APCI–MS analysis of hexane extracts of saponified cell pellets. (n = 3, error bars correspond to standard deviation.)

Two other friedelin synthases have been reported in the literature. *Mi*FRS was identified in the closely related plant *Maytenus ilicifolia* [37] and *Kd*FRS, in the less-related *Kalanchoe daigremontiana* [51]. Accordingly, *Kd*FRS was selected as a surrogate for *Tw*OSC4 for the reconstitution of the early celastrol pathway in yeast, as it was previously shown to produce friedelin in yeast [51]. A synthetic DNA fragment encoding a yeast codon optimized version of *Kd*FRS was cloned into the yeast expression vector pUUS downstream from an inducible promoter and introduced into the strain AM109 (giving rise to strain AM109-3). GC–MS analysis and comparison to an empty vector control strain (AM109-1) showed the appearance of a new constituent with the same retention time and mass spectrum as the authentic friedelin standard (Fig. 6a, b), thus confirming friedelin production in yeast by *Kd*FRS.

### Improving friedelin production by boosting precursor supply

Following establishing friedelin production in yeast by the heterologous expression of *Kd*FRS, we set out to improve friedelin production titers. To this end, different engineered haploid and diploid yeast strains were tested for their ability to produce higher amounts of friedelin. In addition to strain AM109, AM238 was chosen because comparative analysis showed that this strain provided enhanced precursor supply for terpenoid production [52]. AM238 is a haploid strain that contains six deletions, *rox1*, *dos2*, *yer134c*, *vba5*, *ynr063w*, and *ygr259c*, identified in an iterative genetic screen to boost terpenoid production. An additional strain was constructed by further engineering of AM238 to target the regulation of isoprenoid precursor biosynthesis. Specifically, the heterozygous deletion of *UBC7*, a ubiquitin conjugating enzyme part of the ERAD (endoplasmic reticulum associated protein degradation) machinery [53] had previously successfully been used to improve FPP-based terpene biosynthesis in yeast [49]. Thus, we aimed to develop an improved strain for triterpenoid production by deleting the *UBC7* gene from strain AM238 to produce strain AM254, essentially stacking the beneficial mutations.

Initial analysis of the formation of friedelin and its P450 oxygenation products was performed by GC–MS and LC–ESI–MS (electrospray ionization). To more accurately quantify the content of friedelin based on its molecular ion (Additional file 1: Figure S3), a method using atmospheric pressure chemical ionization (LC–APCI–MS) was developed.

The different engineered strains were compared for their ability to produce friedelin using the developed LC–APCI–MS analytic methods. The friedelin titer in strains AM109-3, AM238-2 and AM254-2, each harboring a plasmid expressing *KdFRS* under the control of a galactose inducible promoter (P*GAL1*) were quantified and compared. Empty vector containing cells (AM109-1, AM238-1, or AM254-1) were used as control. After culturing, the cells and spent media were separated by centrifugation and analyzed separately. The pelleted cells were disrupted by saponification and extracted with hexane, while the spent media were directly extracted by hexane. Subsequent analysis of the extracts showed that friedelin was present only inside the cells and not detected in the culture medium. Comparison of the different strains revealed that strain AM254-2 produced approximately 0.5 mg/L of friedelin, 5 times more friedelin than AM238-2 (0.1 mg/L) and 4 times more than AM109-3 (0.12 mg/L; Fig. 6c). Thus, *UBC7* deletion had a profound effect on friedelin productivity and, therefore, strain AM254 was used as a basis for the subsequent polpunonic production efforts.

### Production of polpunonic acid in yeast

Having established friedelin production in yeast, the next step was to synthesize polpunonic acid and other oxygenated fridelanes by co-expressing the three identified P450s in the same system. For catalysis, P450s require cytochrome P450 reductases (CPRs) as a redox partner. While the yeast CPR (Ncp1p) is known to complement heterologous P450s, using plant CPRs usually results in higher activities in yeast [24]. Accordingly, a *T. wilfordii* gene encoding a CPR (*Tw*CPR1) was amplified from cDNA and inserted into the inducible plasmid pESC-LEU. In addition, a constitutive expression plasmid, pHTDH(tHMG2) was included, at it drives the production of an N-terminally truncated form of Hmg2p, known to greatly enhance the heterologous production of triterpenoids in yeast [52]. CYP712K1, 2 or 3 were introduced into this system by transforming with the galactose-inducible expression vectors pESC-TRP(CYP712K1), pESC-TRP(CYP712K2), pESC-TRP(CYP712K3), giving rise to strains AM254-5, AM254-6, and AM254-7, respectively.

**Fig. 7:**
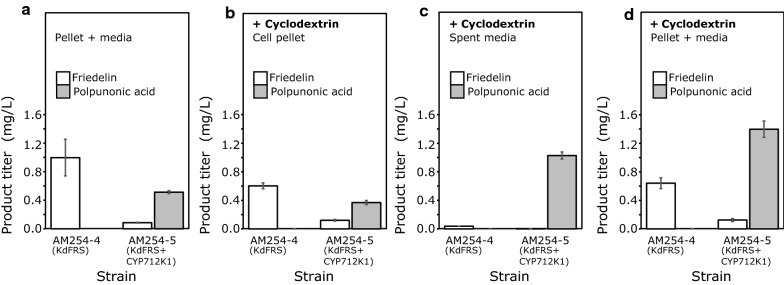
Production of friedelin and polpunonic acid in yeast strains AM254-4 and AM254-5. **a** Total accumulation friedelin (white columns) and polpunonic acid (grey columns) in yeast cell cultures without the addition of cyclodextrins (pellet + media). No friedelin or polpunonic acid were found in the media. **b** Accumulation of friedelin and polpunonic acid in the cell pellets of cyclodextrin-treated cultures. **c** Friedelin and polpunonic acid content in spent media of cyclodextrins-treated cultures. **d** Total content (pellet and media) of friedelin and polpunonic acid in cyclodextrin-treated cultures. (n = 3, error bars correspond to standard deviation)

The combination *Kd*FRS and CYP712K1 (strain AM254-5) resulted in a total (pellet + media) production of ~ 0.5 mg/L polpunonic acid (Fig. 7a). Markedly, friedelin and polpunonic acid were found exclusively inside the cells. Production of polpunonic acid resulted in a significant reduction of intracellular friedelin content to 0.1 mg/L, indicating efficient conversion of the precursor. Expression of the other two P450s, did not produce detectable amounts of polpunonic acid, suggesting that CYP712K2 and CYP712K3 were likely less active or less stable when expressed in yeast under the conditions tested.

In order to further improve the production of polpunonic acid, the effect of addition of cyclodextrins to the yeast media was evaluated. Cyclodextrins are macrocyclic oligosaccharides that consist of 6, 7 or 8 α-1,4-linked glucose moieties. Especially methyl-β-cyclodextrin has previously been shown to stimulate the production of different triterpenoids in yeast [54]. In addition, the use of methyl-β-cyclodextrin in yeast growth media also offers a convenient way to extract triterpenoids from the spent media [54]. The developed strains were grown in the presence of 10 mM methyl-β cyclodextrin to explore whether this would improve polpunonic acid titers. Methyl-β-cyclodextrin did not improve the titer of friedelin when CYP712K1 was not present, rather, a small decrease was observed (Fig. 7b). Friedelin remained mostly intracellular and addition of methyl-β-cyclodextrin sequestered less than 5% of total friedelin in the medium (Fig. 7c). On the other hand, polpunonic acid production was clearly improved in the presence of methyl-β-cyclodextrin. The titer of total polpunonic acid (pellet + media) increased by 2.8-fold, from 0.5 mg/L in the absence to 1.4 mg/L in the presence of methyl-β-cyclodextrin (Fig. 7d compared with Fig. 7a, where no polpunonic acid was found in the media without cyclodextrin) Thus, there was a clear effect of methyl-β-cyclodextrin in the efficiency of polpunonic acid, but not friedelin, export from the cells, since in the presence of methyl-β-cyclodextrin, the majority of polpunonic acid was found in the medium (Fig. 7b, c). The amount of polpunonic acid that remained inside the cells was comparable to the amount found in the pellet in the absence of methyl-β-cyclodextrin (7A), suggesting that extraction of the product by cyclodextrins may also facilitate the turnover of friedelin oxygenation by CYP712K1. Compared to the cells not expressing CYP712K1 (strain AM254-4), strain AM254-5 showed again reduced levels of friedelin (Fig. 7b–d), indicating higher turnover of friedelin to oxygenated products also in the presence of methyl-β-cyclodextrin.

## Discussion

In this study, we developed a platform for the production of polpunonic acid in yeast by integrating enzyme discovery using transient co-expression in tobacco plants with the engineering of a yeast production strain and a cyclodextrin based extraction method. As no genes involved in celastrol biosynthesis in *T. wilfordii* had previously been identified, we started by undertaking a broad gene mining approach to source a catalogue of candidate biosynthetic genes from available transcriptomic data. The first step in celastrol biosynthesis is the cyclization of 2,3-oxidosqualene to produce friedelin by an OSC enzyme. However, enzymes of this class, especially those involved in plant specialized metabolism, are known to often produce more than one products and very rarely can their product profile be predicted from their sequence [33]. Thus, we searched for sequences showing sequence similarity with a well-characterized OSC involved in specialized metabolism, the *A. thaliana* β-amyrin synthase, AtBAS [41] and selected four candidates for further characterization. By transient expression in tobacco, we identified one of the four candidates, *Tw*OSC4, to be a friedelin synthase. While the preparation of this manuscript was in progress, three OSCs from *T. wilfordii* cell suspension cultures (Genbank accession KY885467, KY885468 and KY885469) were published [55]. In agreement with our data, they were found to encode for β‐amyrin, α-amyrin and friedelin synthase activities, respectively.

In triterpenoid biosynthesis, the formation of a triterpene backbone is usually followed by oxidations catalyzed by P450 enzymes [33]. We selected 49 candidate P450s representing 13 different P450 families known to be involved, or related to families involved, in the biosynthesis of terpenoids (Additional file 1: Table S1). Here, a broad choice of candidates proved to be crucial, since the P450s identified to catalyze the first oxidation in celastrol biosynthesis (CYP712K1, CYP712K2 and CYP712K3) are the first enzymes belonging to the CYP712 family to be characterized. This family was included in the selection because it had previously been linked to triterpenoid biosynthesis through gene-cluster association, co-expression analysis and RNAi studies [56–58]. Moreover, the CYP712 family is also phylogenetically related to the CYP705 family, which contains P450s known to be involved in triterpenoid biosynthesis in Arabidopsis [57]. Nevertheless, a CYP712 family enzyme functional in triterpenoid oxidation had not been reported before. Thus, our findings illuminate the function of one more family of plant P450s.

Yeast is generally regarded as a suitable host for enzymes of plant origin [24]. However, several factors, including differences in intracellular pH [59, 60], membrane lipid composition of the ER [30, 31] (relevant for ER-membrane-anchored OSCs and P450s), and availability of dedicated co-factors, such as, for example, specialized cytochrome b5 enzymes [32], are generally regarded to cause a plant enzyme not to function properly in a yeast cell. To minimize the risk of failing to identify enzymes in the celastrol pathway because of suboptimal conditions, we performed functional screening of enzymes by transient co-expression in tobacco. This approach proved successful, as the identified friedelin synthase, *Tw*OSC4, failed to produce friedelin when expressed in yeast. The usefulness of this approach was also demonstrated by the fact that when CYP712K2 and CYP712K3 were co-expressed with a friedelin synthase, they were able to produce polpunonic acid in tobacco but not in yeast. The inability to observe friedelin oxygenation by CYP712K2 and CYP712K3 in yeast could be due to differences in translational efficiency, mRNA or protein stability. A possible downside of the approach can be the ability of endogenous tobacco enzymes to further modify the enzymatic products. For example, it is established that tobacco often glycosylates products of heterologous enzymes resulting in a multitude of glycoside products [61]. This can be circumvented by the use of glycosylase formulations, such as Viscozyme (Sigma-Aldrich) to deglycosylate the compounds to their original form prior to analysis.

The three CYP712K enzymes that we identified were all able to convert friedelin to two new compounds, 29-hydroxy-friedelin and polpunonic acid, when co-expressed with *Tw*OSC4 in tobacco. This stepwise oxidation reaction likely follows the same pattern for carboxylic acid formation as previously observed in the well-characterized stepwise oxidation of β-amyrin to oleanolic acid [45]. First, oxidation of the methyl group to an alcohol, then to the corresponding aldehyde, and finally oxidation to a carboxylic acid. This pattern has previously been described for the oxidation of carbons 23, 24, 28, and 30 of an oleanane backbone [45, 62–65]. Although all three CYP712Ks identified here oxidize the C-29 position on friedelin, we noted that each of them produced a different ratio of 29-hydroxy-friedelin to polpunonic acid (Fig. 3). CYP712K1 appears to produce higher absolute amounts of polpunonic acid and exhibits a higher polpunonic acid to 29-hydroxy-friedelin ratio than CYP712K2 or CYP712K3. This type of differences in the efficacy of conversion of the intermediate alcohols by P450s has previously also been observed in the case of α-amyrin, β-amyrin and lupeol C-28-oxidizing CYP716As [45, 46, 66].

In addition to friedelin synthase (*Tw*OSC4), we also identified three other OSC enzymes. Two of these, *Tw*OSC1 and *Tw*OSC2, produce mainly β-amyrin, while the third one, *Tw*OSC3, produces α-amyrin. Triterpenoid compounds derived from β-amyrin, such as oleanolic acid, are widespread across most orders of higher plants [67]. In *T. wilfordii*, several oleanane type triterpenoids, many of which contain a carboxylic acid group at the C-29 position [68], have been reported, suggesting that these decorations could be formed by either CYP712K1, CYP712K2 and CYP712K3, or highly similar enzymes. Here, we show that CYP712K1 and CYP712K2 can turn products of *Tw*OSC2 and *Tw*OSC3 into the new compounds, **7**, **8** and **9** (Additional file 1: Figure S2). Although we did not confirm the structure of these compounds directly, we speculate that **7** is 29-hydroxy-β-amyrin and **8** is 29-carboxy-β-amyrin, because they are produced only when the P450s are co-expressed with the two β-amyrin producing OSCs (*Tw*OSC1 and *Tw*OSC2). In addition, we hypothesize that **9** likely corresponds to 29-carboxy-α-amyrin because it is only observed when CYP712K1 or CYP712K2 are co-expressed with the predominantly α-amyrin producing *Tw*OSC3. Moreover, we observe that CYP712K1 and CYP712K2 have different apparent substrate specificities for α-amyrin and β-amyrin (Additional file 1: Figure S2). In combination with the β-amyrin-producing *Tw*OSC2, CYP712K1 produces mostly **7**, while CYP712K2 mostly **8**. However, when both CYPs are present we observe mostly **8**, suggesting that **8** (putative 29-carboxy-β-amyrin) may be a derivative of **7** (putative 29-hydroxy-β-amyrin). According to this, it appears that CYP712K2 catalyzes efficiently all the steps until the formation of 29-carboxy-β-amyrin, whereas CYP712K1 produces mostly 29-hydroxy-β-amyrin. By contrast, when using friedelin as substrate, CYP712K1 produces mostly polpunonic acid, while CYP712K2 produces mostly 29-hydroxy-friedelin (Fig. 3). Thus, these findings suggest that friedelin is likely the preferred substrate for CYP712K1, while β-amyrin is likely the preferred substrate for CYP712K2. In addition, we observed that expression of CYP712K2 together with the α-amyrin-producing *Tw*OSC3 resulted in the production of **9** (putative 29-carboxy-α-amyrin), whereas co-expression of CYP712K1 with *Tw*OSC3 did not produce this compound. This observation suggests that CYP712K1 and CYP712K2 likely also have different preference for α-amyrin.

Having identified a friedelin synthase and three P450s capable of performing what appears to be the first oxygenation step in celastrol biosynthesis, we set out to produce polpunonic acid in yeast. As *Tw*OSC4 proved to be inactive when expressed in yeast, *Kd*FRS was chosen as a surrogate friedelin synthase because it had previously been shown to be functional in yeast [51]. Experiments with *Kd*FRS yielded a maximum titer of 0.12 mg/L of friedelin. This number is in line with the friedelin titers previously reported using non-engineered production strains (0.05 mg/mL with *Kd*FRS; 0.1 mg/mL with *Mi*FRS) [38]. Compared with the yields of other triterpenoids [69], these titers are relatively low. We interpret this as the result of inherent properties of friedelin itself, such as potential toxicity to yeast, or the mechanism of friedelin production by *Kd*FRS, as friedelin is the last product of a long and complicated cycloisomerization cascade [33]. However, the recently published work of Zhou et al. [55], reports a titer of more than 2 mg/L from expressing KY885469 in strain BY4741 [70]. This suggests that culture conditions or extraction method may be responsible for the differences in the reported yields and indicates that there is potential for important further improvement of friedelin production titers.

In order to increase friedelin production in yeast three different yeast strains, including AM109 [49], AM238 [52] and AM254 (created in this study) were evaluated. AM254 was derived from AM238 by the deletion of *UBC7* [71], that encodes for an E2 ubiquitin conjugating enzyme active in the yeast ERAD [72, 73]. Ubc7p is needed for the ubiquitination of some membrane proteins, such as the sterol biosynthesis bottleneck enzyme Erg1p (squalene epoxidase) by the E2 ubiquitin ligase Doa10p [74], and for the feedback-regulated degradation of the key regulatory enzymes of the sterol pathway, Hmg1p and Hmg2p [53]. Thus, the friedelin titer improvements observed as a result of *UBC7* deletion are likely due to elevated levels of Erg1p, Hmg1p, and Hmg2p, resulting in increased flux to 2,3-oxidosqualene. Nevertheless, the possibility that *UBC7* deletion may also have additional effects, such as, for example, friedelin synthase stabilization, cannot be ruled out.

We established polpunonic acid production by co-expression of *Kd*FRS, tHMG2, CYP712K1 and *Tw*CPR1 in the strain AM254-5 (Fig. 7a). Friedelin and polpunonic acid were found exclusively in the pellet and not in the spent media. To further improve the production of friedelin and polpunonic acid and to introduce a convenient way of extracting these triterpenoids from spent yeast media, β-methyl-cyclodextrin was added to the yeast cultures. This method omits the chemical or mechanical homogenization of cells and, thereby, considerably shortens the time required for sample preparation. Furthermore, as only the medium is extracted and not the whole cell content, it results in significantly lower background. Cyclodextrins are known to facilitate triterpenoid production leading to significant improvements in triterpenoid titers [54]. However, the exact mechanism by which cyclodextrins facilitate the excretion of triterpenoids is not yet known. In our experiments, adding cyclodextrins to the media did not have an apparent effect on total friedelin titers and friedelin was still found mostly in pellet and to a lesser extend in the media. However, the total polpunonic acid titer increased 2.8-fold compared to the cells not treated with cyclodextrin. In these cells, polpunonic acid was found mostly in the spent media (71%) and to a lesser extent (29%) in the cell pellet. This is well in line with a previous report where cyclodextrins were used to improve the production of the oxidized triterpenoid, 16-hydroxy-β-amyrin [54]. The different effect of cyclodextrins on friedelin and polpunonic acid accumulation could possibly be related to the different functional groups and polarity of the two compounds. Perhaps the carboxyl group on polpunonic acid contributes to hydrogen bonding with the OH-groups of cyclodextrins. Alternatively, the presence of the carboxyl group may play a role in the interaction of polpunonic with the plasma membrane, which may be a critical factor for its extraction by cyclodextrins.

In summary, we characterized four oxidosqualene cyclases, *Tw*OSC1 as a β-amyrin synthase, *Tw*OSCC2 as a mixed β-amyrin and lupeol synthase, *Tw*OSC3 as a mixed α-amyrin and β-amyrin synthase and *Tw*OSC4 as friedelin synthase. We also characterized CYP712K1, CYP712K2 and CYP712K3 as triterpenoid oxidizing enzymes acting on C-29. These enzymes catalyze the sequential formation of 29-hydroxy-friedelin, 29-oxo-friedelin, and polpunonic acid, and most likely the corresponding oxidations of α- and β-amyrin, and are the first members of the CYP712 family to be reported to function in terpenoid metabolism. Having elucidated the cyclization and first triple-oxidation step of celastrol biosynthesis, we established a platform for the production of polpunonic acid in yeast. These results pave the way for the discovery of the missing enzymes in celastrol biosynthesis and provides an important advancement in the efforts to produce celastrol using biotechnological methods.

## Conclusions

In this study, we used a combination of database mining with functional screening of candidate genes in tobacco to elucidate missing steps of the celastrol biosynthetic pathway. The newly discovered enzymes enabled the synthesis of polpunonic acid in engineered yeast. These advances pave the way for the complete elucidation of celastrol biosynthesis and the production of this potent anti-obesity agent in microbial cell factories.

## Methods

### Plant material

*Tripterygium wilfordii* plants were obtained from Plantentuin Esveld (NL) and grown under greenhouse conditions at a day length of minimum 11 h and a temperature of minimum 12 °C. Samples of root and leaf tissue where harvested and immediately ground in liquid nitrogen, then stored at − 80 °C until further use for metabolite analysis and RNA isolation.

### Root transcriptome mining using the MassBlast tool

The root transcriptome previously described by Hansen et al. [39] was mined using MassBlast, version 0.9.9 for win-32 [40]. MassBlast identifies and ranks putative homologs of entries in a custom-made query database. Separate query databases were used to identify candidate OSCs (the query database file contained only accession BAG82628; *At*BAS) and candidate P450s (the query database was the P450 reference database of Zerbe et al. [43]). In the user.yml file, “engine” was set to tblastn, “-evalue” set to 1, “identity: min:” set to 0.35, “prune_identical: list:” set to—nt_db_longest_orf and otherwise default settings. The output tables were subsequently manually filtered and sorted.

### Cloning

Transcript specific primers for candidate OSCs were used in an initial amplification from cDNA (SuperScript^®^ III First-Strand Synthesis System; ThermoFisher; Catalog No. 18080051) prepared from in house *T. wilfordii* root RNA (Spectrum Plant Total RNA Kit; Sigma Aldrich; product # STRN50). All possible inter-contig combinations of forward and reverse primers were in addition tested. Gel purified (QIAquick Gel Extraction Kit; QIAGEN; Catalog No. 28706) amplicons of expected size were subsequently used as templates for amplifications using primers designed for USER cloning. Resulting amplicons of expected size were gel purified and cloned, using USER cloning, into the USER compatible destination vector, pCAMBIA-1300-35Su (encoding kanamycin resistance), previously used for transient gene expression in tobacco [42]. Primers are listed in Additional file 1: Table S1.

Transcript specific primers of candidate P450s were used in an initial amplification from root derived cDNA except for seven candidates that were amplified from leaf cDNA (Additional file 1: Table S1). After blunt end ligation of gel purified amplicons of expected size into pJET1.2/blunt (Thermo Fisher Scientific Inc.; Cat# K1231), resulting constructs were used as template for another amplification using primers designed for USER cloning. Gel purified amplicons of expected size were USER-cloned into pCAMBIA-1300-35Su. Some constructs were directly obtained by amplification from cDNA with USER compatible primers (indicated in Additional file 1: Table S1).

Yeast expression constructs were produced with USER cloning or restriction enzyme cloning of candidates and other parts into the yeast expression vectors pESC-LEU or pESC-TRP (Agilent Technologies, Cat. # 217452 and 217453) or pUUS (pESC-URA-USER) [75] according to Additional file 1: Table S2. Coding sequences for CYP712K1, CYP712K2, CYP712K3, *Tw*OSC1, *Tw*OSC2, *Tw*OSC3, *Tw*OSC4 and *Tw*CPR1 were deposited in GenBank under accession numbers MN621243–MN621250, respectively.

### Transient co-expression in tobacco leaves

*Tripterygium wilfordii* OSCs and/or candidate P450s were transiently (co-)expressed in tobacco leaves, together with the gene silencing suppressor p19 [76] and the *Coleus forskohlii* 1-deoxy-d-xylulose 5-phosphate synthase (*Cf*DXS; elevating isoprenoid diphosphate precursor pool [27, 77]), essentially according to a previously established protocol [78]. Briefly, harvested cultures of transformed *Agrobacterium tumefaciens* strain AGL-1 [78] were adjusted to OD_600_ = 1 in water and mixed 1:1 in respective gene combinations before infiltrated. Metabolites were extracted 6–8 days post infiltration from two leaf discs (Ø3 cm, 1 disc per leaf) per gene combination in a desired solvent (see analytical procedures for details) and analyzed. As background controls, p19 and *Cf*DXS were co-expressed alone. Plant material for purification of compounds **5** and **6** were prepared by large-scale vacuum infiltrations (48 plants per compound) as previously described [79]. Overnight cultures (15 mL) of transformed *A. tumefaciens* were used as starter cultures for subsequent cultures of 200 mL. These were grown overnight to OD_600_ > 1 before harvested, adjusted to OD_600_ = 1 using water, and mixed in equal volumes to obtain the respective gene combinations prior to infiltration. Infiltrated leafs were harvested 8 days post infiltration and stored at − 20 °C until further used.

### Extraction and isolation of compounds 5 and 6

Tobacco leaves containing compounds, **5** and **6** were harvested, ground in liquid nitrogen and extracted three times with 200 mL of ethyl acetate (1 h of shaking for each). Total crude ethyl acetate extracts were dried by rotor evaporation. Compound **5** was separated by repeated Silica gel column chromatography eluted with CH_2_Cl_2_:MeOH (95:5), and further purified by Sephadex LH-20 (MeOH). In a resulting fraction, compound **5** formed crystals that were washed to purity with hexane. For isolation of **6**, the dry residue was subjected to repeated silica gel column chromatography successively eluted with CH_2_Cl_2_:acetone (98:2, 9:1, 4:1, 1:1, 0:1) and acetone:ethyl acetate (9:1, 1:1, 0:1). In a resulting fraction, compound **6** formed crystals that were washed to purity in acetone (final yield = 2.5 mg).

### Extraction of *T. wilfordii* root and leaf metabolites

Plant tissue (25–50 mg) was ground in liquid nitrogen, transferred to a glass vial, and extracted with 1 mL methanol for 1 h with shaking at RT. The residue was spun down and the supernatant diluted 1:5 with methanol before analyzed with LC–ESI–MS. Plant tissues were extracted and analyzed in three biological replicates (i.e. from three individual plants).

### Construction of yeast strains and yeast expression of candidates

To create strain AM254, a disruption cassette for *UBC7* in strain AM238 was created. A large PCR reaction (300 μL) was prepared using vector pUG27 as template, MyTaq™ DNA Polymerase (Bioline) and the primers UBC7 pUG-F: and UBC7 pUG-R (Additional file 1: Table S1). PCR cycling conditions were: initial denaturation at 95 °C for 1 min followed by 35 cycles of denaturation at 95 °C for 30 s, annealing at 52 °C for 30 s and extension at 72 °C for 1 min and 1 cycle of extension at 72 °C for 5 min. The amplicon was gel purified and precipitated with phenol/chloroform extraction, chloroform extraction (twice), ethanol precipitation, and finally air dried. Strain AM238 was transformed with the pelleted amplicon as described above and the transformation mixture was plated on Glu/CM-His agar for selection and incubated for 3 days at 30 °C. Single colonies of the transformants were gridded in a new Glu/CM-His agar plate. Genomic DNA was extracted from 50 mL overnight cultures originating from eight different yeast colonies. PCR was performed for verification of cassette integration with MyTaq™ DNA Polymerase (Bioline), gDNA as template and the primers UBC7_pUG_R and UBC7_prom_F. Five correct clones were identified and verified by sequencing, and one of these was carried forward to excise the loxP-his-loxP fragment by yeast transformation with the Cre recombinase-expressing plasmid pB227/Gal-Cre and growth in Gal-Raff/CM-Leu agar broth. Cells cured for the plasmid were tested for loss of HIS5 marker by PCR, thereby generating strain AM254.

The yeast strains listed in Table 1 were produced by transforming the parent strains with yeast expression plasmids using the lithium acetate method [80]. For the production of friedelin and derivatives, pre-cultures of the strains were grown until saturation in synthetic defined media including 2% glucose. Then, the cells were washed with water and inoculated into 10 mL synthetic defined media with 2% galactose and 1% raffinose with or without 10 mM β-methyl cyclodextrins and grown for 96 h at 30 °C and 150 rpm.

### Extraction of triterpenoids from yeast cells and media

Cell pellets from 10 mL yeast cultures were disrupted by saponification by addition of 10 mL 20% KOH/25% ethanol and then incubated for 1 h at 90 °C. Subsequently the saponified cells were extracted three times with 5 mL of hexane.

### Compound extraction from tobacco leaves

One leaf disc (Ø3 cm) was ground in an Eppendorf tube and mixed with 0.6 mL 50 mM sodium acetate, pH 5.3. To this, 20 µL of Viscozyme (Sigma-Aldrich) or water (control) was added prior to shaking for 1 h at room temperature. The Viscozyme treated sample appeared viscous compared to control. Metabolites were extracted in 1 mL ethyl acetate by shaking for 5 min and the upper layer was transferred to new vials. The solvent was evaporated under a stream of nitrogen and the residue re-suspended in 1 mL methanol and analyzed by LC–ESI–MS.

### Analytical procedures

GC–MS analyses of non-derivatized samples were performed on a Shimadzu GC–MS-QP2010 Ultra fitted with an Agilent HP-5MS column (Length: 20 m; Thickness: 0.18 µm; Diameter: 0.18 µm). The injection volume was 1 µL at 250 °C (splitless mode) and the GC program was as follows: 60 °C for 1 min, ramp at the rate of 30 °C/min to 260 °C, ramp at the rate of 5 °C/min to 300 °C, ramp at the rate of 30 °C/min to 320 °C, hold for 3 min.

Alternatively, GC–MS analysis was carried on a DB-5 column using the following program: Injection volume was 2 µL. The initial temperature 60 °C, ramp to 300 °C with a rate of 10 °C/min, ramp to 320 °C with a rate of 3 °C/min, hold for 3.5 min.

Sample derivatization with trimethylsilyl cyanide was automated using a GERSTEL MultiPurpose Sampler (MPS) with DualRait WorkStation integrated to a GC–MS system from Agilent (7890A GC and an Agilent 5975C series MSD). Separation of derivatized samples was achieved using an Agilent HP- 5MS column (30 m × 250 μm × 0.25 μm) with hydrogen as carrier gas at a constant flow rate of 1.2 mL/min. The GC oven temperature program was as follows: initial temperature, 60 °C; equilibration time, 1 min; heating rate, 12 °C/min; end temperature, 310 °C; hold time, 6 min; and post-run time, 5 min at 60 °C.

Qualitative LC–ESI–MS analysis was performed on the Dionex UltiMate^®^ 3000 Quaternary Rapid Separation UHPLC focused system (Thermo Fisher Scientific, Germering, Germany) equipped with a Phenomenex Kinetex XB-C18 column (100 mm × 2.1 mm i.d., 1.7 µm particle size, 100 Å pore size) (Phenomenex, Inc., Torrance, CA, USA). The column was operated at 40 °C, and the flow rate was maintained at 0.3 mL/min. The mobile phases were water (A) and 100% acetonitrile (B), both acidified with 0.05% formic acid. Separations were performed using the following gradient profile: 0 min, 37% B; 11 min, 80% B; 21 min, 90% B; 22 min, 100% B; 27 min, 100% B; 28 min, 37% B. The column outlet was connected to a Bruker Daltonics Compact QqTOF mass spectrometer equipped with electrospray ionization (ESI) interface (Bruker Daltonics, Bremen, Germany). Mass spectra were acquired in positive ion mode, using a capillary voltage of 4500 V, an end plate offset of − 500 V, a drying temperature of 250 °C, a nebulizer pressure of 1.2 bars, and a drying gas flow of 8 L/min. Sodium formate solution (internal standard) was injected at the beginning of each chromatographic run and the LC–MS raw data was calibrated against these sodium clusters using the Data Analysis 4.1 (Bruker Daltonics) software program. Quantitative LC–APCI–MS was performed with the same equipment and program except that atmospheric pressure chemical ionization (APCI) interface (Bruker Daltonics, Bremen, Germany) was used instead and mobile phase (B) was 50% acetonitrile–50% methanol with 0.05% formic acid.

### Standards

Celastrol, α-amyrin, β-amyrin, lupeol, betulinic acid, hederagenin, oleanolic acid and friedelin were purchased from Sigma-Aldrich.

### NMR analysis

All NMR spectra were recorded at 300 K in CDCl_3_. NMR experiments for compound **5** and **6** were performed with a 600 MHz Bruker Avance III NMR equipped with a 5 mm broad band probe (Bruker Biospin) optimized for ^1^H and ^13^C. All experiments were acquired in automation (temperature equilibration to 300 K, optimization of lock parameters, gradient shimming, and setting of receiver gain). One-dimensional ^1^H and ^13^C NMR spectra were acquired with 30°-pulses and 64 k data points. IconNMR ver.4.2 (Bruker Biospin, Karlsruhe, Germany) was used for controlling automated sample change and acquisition of NMR data, whereas TopSpin ver. 3.5 (Bruker Biospin, Karlsruhe, Germany) was used for acquisition and processing of NMR data.

## Supplementary information

**Additional file 1: Table S1.** Clone and primer list. **Table S2.** Yeast expression constructs. **Table S3.** Assigned 1C and 13C NMR peaks for compound 5 and 6. **Figure S1.** Comparison of the mass spectra of TMS derivatized *Tw*OSC products from tobacco leaf co-infiltration experiments, with TMS derivatized authentic standards. **Figure S2.** CYP712K1 and CYP712K2 oxygenate the triterpene products of *Tw*OSC2 and *Tw*OSC3 in tobacco. Two leaf discs (Ø3 cm) of leaves co-expressing the indicated enzymes were extracted in 1 mL ethyl acetate. A 0.15 mL aliquot of this extract was dried, TMS derivatized and analyzed by GC–MS. None of the novel peaks **7-9** had mass spectra or retention times matching any of the analyzed authentic standards (betulinic acid, hederagenin, or oleanolic acid; not shown). **Figure S3.** Comparison of LC–ESI–MS and LC-APCI-MS (base peak chromatograms) for the reliable quantification of friedelin and polpunonic acid. Authentic standards of friedelin and polpunonic acid (30 µg/mL) were analyzed using the same LC–MS equipment and program with exception of ionization probe (ESI; black trace and APCI; red trace).

## Data Availability

The datasets used and/or analyzed during the current study are available from the corresponding author on reasonable request.
