# Supplementary material for: Integrating pathway elucidation with yeast engineering to produce polpunonic acid the precursor of the anti-obesity agent celastrol
Source: Microb Cell Fact. 2020 Jan 28;19:15. doi: 10.1186/s12934-020-1284-9 (PMC6988343; doi:10.1186/s12934-020-1284-9)
Supplement: Supplementary file 1 — Additional file 1: Table S1. Clone and primer list. Table S2. Yeast expression constructs. Table S3. Assigned 1C and 13C NMR peaks for compound 5 and 6. Figure S1. Comparison of the mass spectra of TMS derivatized TwOSC products from tobacco leaf co-infiltration experiments, with TMS derivatized authentic standards. Figure S2. CYP712K1 and CYP712K2 oxygenate the triterpene products of TwOSC2 and TwOSC3 in tobacco. Two leaf discs (Ø3 cm) of leaves co-expressing the indicated enzymes were extracted in 1 mL ethyl acetate. A 0.15 mL aliquot of this extract was dried, TMS derivatized and analyzed by GC–MS. None of the novel peaks 7-9 had mass spectra or retention times matching any of the analyzed authentic standards (betulinic acid, hederagenin, or oleanolic acid; not shown). Figure S3. Comparison of LC–ESI–MS and LC-APCI-MS (base peak chromatograms) for the reliable quantification of friedelin and polpunonic acid. Authentic standards of friedelin and polpunonic acid (30 µg/mL) were analyzed using the same LC–MS equipment and program with exception of ionization probe (ESI; black trace and APCI; red trace). [file 12934_2020_1284_MOESM1_ESM.pdf]

Additional file 1

Table S1. Clone and primer list.

X = deoxyuracil

F = forward primer

R = reverse primer
UF = USER compatible forward primer
UR = USER compatible reverse primer
leaf = amplified from leaf from leaf derived cDNA

Clones only listed with UF and UR, were amplified from cDNA using these primers

Clones list with F, R, UF and UR, were amplified from cDNA using F and R primers

Type	Gene	Family	Clan	Primer type	Sequence
**OSCs**	OSC1			F	ATGTGGAGGCTAAAGATTGCAGAGG
				R	CTGCTAGACTTTGGAAGATAATGTTACTCTC
				UF	GGCTTAAXATGTGGAGGCTAAAGATTGCAGA
				UR	GGTTTAAXGCTAGACTTTGGAAGATAATGTTACTCTC
	OSC2			F	ATGTGGAGGCTAAAGATTGCAGAGG
				R	TTAGGCATCAGAAGGTAGAGGAAGCAG
				UF	GGCTTAAXATGTGGAGGCTAAAGATTGCAGA
				UR	GGTTTAAXTTAGGCATCAGAAGGTAGAGGAA
	OSC3			F	ATGTGGAGGCTTAAGGTGGGCGA
				R	GGACTGGGCATTGGTCTAGCATTTAC
				UF	GGCTTAAXATGTGGAGGCTTAAGGTGGG
				UR	GGTTTAAXTGGGCATTGGTCTAGCATTTAC
	OSC4			F	ATGTGGAAGATAAAGATAGCTGAACGTGG
				R	TCATTGCAATTTAATTTCCTTTGAAGGCAACT
				UF	GGCTTAAXATGTGGAAGATAAAGATAGCTGAACG
				UR	GGTTTAAXTCATTGCAATTTAATTTCCTTTGAAGGC
**CYPs**	CYP712K1	CYP712	71 clan	F	CCTTCTTCGCAATCTCCAAT
				R	CCTTAGTTTTCTGCTTAACCG
				UF	GGCTTAAXATGGCCACCATCACTGACATCCAATACT
				UR	GGTTTAAXTTAACCGGCAAATGGATTGAAACGAACGACCGG
	CYP712K2	CYP712	71 clan	F	GCCTAAACCACTCTAATAAGCCA
				R	AACCACAGCTTATCTCCAACCATC
				UF	GGCTTAAXATGGCCACTACCATCATTGACGTCCAAT
				UR	GGTTTAAXTTAGGAAGAAAATGGATCAAATCGGACGATTGG

(Table S1 continued)

Type	Gene	Family	Clan	Primer type	Sequence
	CYP712K3	CYP712	71 clan	F	CGATAAGCCTAAACCACTCTAATAAGCCA
				R	GCAAGAAAAGGGATGGAATCGGAGA
				UF	GGCTTAAXATGGCCACCACTACCATCATTGAC
				UR	GGTTTAAXTTAGCAAGAAAAGGGATGGAATCGGAGA
	CYP71AN56	CYP71	71 clan	F	ATGGATCTTGTTATGTTAATGCATAAATCAAATCAATTTTATGCCATCTCTGTC TCTTTG
				R	GAGGAGATCAATGAGAATGC
				UF	GGCTTAAXATGGATCTTGTTATGTTAATGCATAAAT
				UR	GGTTTAAXTTAATGAGAATGCATTAGTGGGACAAGATTAAG
	CYP71AP40	CYP71	71 clan	F	TAATGCTGCCTTTCCTTCAGT
				R	GGTGATTTATTGTCTTAGGGG
				UF	GGCTTAAXATGCTGCCTTTCCTTCAGTTGCTGATCC
				UR	GGTTTAAXTTAGGGGAACCATGGTTTGGCAACAACAATCAG
	CYP71B102	CYP71	71 clan	F	AGACGTAGCATAGTACCATTC
				R	GTGTATCTACGAGTAACTTCAGC
				UF	GGCTTAAXATGGCTCTGCATACCTTAGCAA
				UR	GGTTTAAXTTAGCTTGTGTTATTTACTGGTAATGAC
	CYP71E23	CYP71	71 clan	F	TACACGGCTCACTTCTTGTT
				R	ACTTGATACTTCCACCGTATG
				UF	GGCTTAAXATGGCCATCATTTCACAACAA
				UR	GGTTTAAXTTAACAGAGCCCTATATCCTTCG
	CYP71BE86	CYP71	71 clan	F	CAGTAACCACTCACACCTATCTC
				R	CCAGTATGGAACCTTGTGCTTG
				UF	GGCTTAAXATGGATCTCCAACTCCCGTCCTTCCCGA
				UR	GGTTTAAXTTAACCGGAGAGCGAGCGATAAGGAGTTGGAAC
	CYP71BE85v1	CYP71	71 clan	F	CCTCATGGACCTCCTACAAT
				R	AAGGGAGATAACACTGCTAC
				UF	GGCTTAAXATGGACCTCCTACAATTTCCATCGGTCT
				UR	GGTTTAAXTTAGTTAAAGGCAGGTGAAGGACAATAAGGAGT
	CYP71BE84	CYP71	71 clan	F	CCATCATGGATCATCACCTTACT
				R	GTCGTAATATAGGGGAACATCAC
				UF	GGCTTAAXATGGATCATCACCTTACTTCTTTTCCAC
				UR	GGTTTAAXTTACTTGTTTTCCTCAACAACAGAGGACTTATA
	CYP71BE83	CYP71	71 clan	F	TCATCATGGATCTTCAGTTTTC
				R	CACTCTTAGTTTCATTCACTCG
				UF	GGCTTAAXATGGATCTTCAGTTTTCTTCTTTTCCGG
				UR	GGTTTAAXTTACTCGACAATTTTGGGATGATAAGGAATTGG
leaf	CYP71BE85v2	CYP71	71 clan	UF	GGCTTAAXATGGACCTCCTACAATTTCCATCGGTCT
				UR	GGTTTAAXTTAGTTAAAGGCAGGTGAAGGACAATAAGGAGT
	CYP76T30	CYP76	71 clan	F	AGGAAGTGCTAGGAACAGAC
				R	CACACATGCATTGCCATTTAG
				UF	GGCTTAAXATGGACTACTTTACGCTATTAGCGCTGA
				UR	GGTTTAAXTTATACTTGGATAGGGACAGCCAGGAGAGGAAG

(Table S1 continued)

Type	Gene	Family	Clan	Primer type	Sequence
	CYP76Y20	CYP76	71 clan	F	CCTTCCATGCTCTCATCCATAC
				R	ATTTGCACACACCATTGCT
				UF	GGCTTAAXATGGAACTTCACATCTTCCTTTTCTGCC
				UR	GGTTTAAXTTAATTGTTATTGGCTTGGATGGGGATAGCACA
	CYP711A62	CYP711	711 clan	F	GCGAATGTTCCTGCTACTATCTTCA
				R	CACTACTCTAAGCTTTACACCATGCCT
				UF	GGCTTAAXATGGAGTTAGTGGGTGTAGAGTTTTTC
				UR	GGTTTAAXTCATTCTAGAAAGATCACTACTCTAAGCTTTACA
	CYP81AM1	CYP81	71 clan	F	CATGGAAACCCTTCACTACTTGGGTC
				R	AGTGTGCACACACACGCAGATA
				UF	GGCTTAAXATGGAAACCCTTCACTACTTGGGTCTCT
				UR	GGTTTAAXTTATAGGTGGGAAAGTGCGGCGGTGAGAGGCTG
	CYP82C61	CYP82	71 clan	F	GCTCTCCAGATGGAGTTTCTTC
				R	ACAAACCAGCAACACAAAGTAAAC
				UF	GGCTTAAXATGGAGTTTCTTCTTTCACTTCCAACAA
				UR	GGTTTAAXTTACCCCATATAGAGATGACTTGGAAGGCGTGG
	CYP82AS1	CYP82	71 clan	F	GTCACATGCTTTGCTTCCGTA
				R	CTCCTTAAGCAGAAAACAGTAACA
				UF	GGCTTAAXATGCTTTGCTTCCGTAGCCCCAAGCCCT
				UR	GGTTTAAXTTAGTGTACGTAAAGCTTAGGATCAAGACGCGG
	CYP82D166	CYP82	71 clan	F	CTGTTACAATGATTCCCCTTCTGC
				R	TGACGGTAAGGAATAGCCCTAGGT
				UF	GGCTTAAXATGATTCCCCTTCTGCCATACCTGAATA
				UR	GGTTTAAXTTATTCATAGGCAGTAGGAGACAAGCGAGGTCT
	CYP82D169	CYP82	71 clan	F	GCTGCAACCATGGAGTACTCAA
				R	AACACAAATGGCAAGTGGCAACA
				UF	GGCTTAAXATGGAGTACTCAATTACCACCATTGGAG
				UR	GGTTTAAXTTAATTATAAAGGTGGGTAGGGAGGCGAGGCGA
	CYP82C60	CYP82	71 clan	F	TAACCATCAGCACTCCTGT
				R	CTCCTCTCTCAAACCACAT
				UF	GGCTTAAXATGTCCATACACTTACAGCTCACAACAA
				UR	GGTTTAAXTTATCCATATAAATGAGGTGGGAGACGTGGACT
	CYP82J13	CYP82	71 clan	F	CTCACGATCATGGATTTCCC
				R	TTTTCCCTTCCAATTTAGCTT
				UF	GGCTTAAXATGGATTTCCCTTCCAATCTAACAGCAA
				UR	GGTTTAAXTTAATAGAGATCAGAACAAAGTCTTGGACTGAC
	CYP82D168	CYP82	71 clan	F	CTAGGCAATAGAAACCATGGAATC
				R	TCCTTAACGTAGGCATATTGACAC
				UF	GGCTTAAXATGGAATCTCTTCTCCTCCTCCAGGTAC
				UR	GGTTTAAXTTAATCATACAGGTGGGCTGGGTGGCGTGGACT
leaf	CYP82AQ1	CYP82	71 clan	UF	GGCTTAAXATGGATTTTGCGCTACCATTGCCCACAA
				UR	GGTTTAAXTTAAGCATAAAGGTGGGCAGGAAGGCGTGGAGT

(Table S1 continued)

Type	Gene	Family	Clan	Primer type	Sequence
leaf	CYP82D167	CYP82	71 clan	UF	GGCTTAAXATGGCAGACAAGTGCGGCCCAATCTTCA
				UR	GGTTTAAXTTAGGTGCTAGTACTCCAATTATCCATATAGAG
	CYP701A58	CYP701	71 clan	F	ATGGCGGCTCCAACTCCTTCT
				R	TCAATTTCTTGGCTTTATCATTGCGTGCAAC
				UF	GGCTTAAXATGGCGGCTCCAACTCCTTCT
				UR	GGTTTAAXTCAATTTCTTGGCTTTATCATTGCGTGCAAC
	CYP72D19	CYP72	72 clan	F	ACATGGAAGGCTTGATTGTG
				R	CCTCAAAGCGGGTGAAGGATAA
				UF	GGCTTAAXATGGAAGGCTTGATTGTGCAAACGATGT
				UR	GGTTTAAXTTAAAGCGGGTGAAGGATAATGGGAGCTCCATG
	CYP76A55	CYP72	72 clan	F	TCCGCTTGCTCCAGAATGGTTT
				R	TGGGGTAGGCCACACAATCAT
				UF	GGCTTAAXATGGTTTGCCTCTGTAGTTGGTTTGTTT
				UR	GGTTTAAXTTACATGATTCGTTTCTTTGGTATTAGTTTCAA
	CYP711A61	CYP711	711 clan	F	CATGGAATTTGTGGGTGTAACTGTG
				R	TCATGTTCTATTCACTACTCTAAGCTTTACAC
				UF	GGCTTAAXATGGAATTTGTGGGTGTAACTGTGA
				UR	GGTTTAAXTCATGTTCTATTCACTACTCTAAGCTTTACAC
	CYP749A88	CYP749	72 clan	F	CCAGTCATGAGCTTAGTAACCC
				R	CAGAGTGACCAATTTAGCCCTTTTA
				UF	GGCTTAAXATGAGCTTAGTAACCCTAATTGTTGGTC
				UR	GGTTTAAXTTAATTCCCATTATCCAACCGTTGAAGGATAAT
	CYP749A89	CYP749	72 clan	F	GCAATGGCCAGTTTAGGGA
				R	CCCAATACAAACTCAAAATGCAG
				UF	GGCTTAAXATGGCCAGTTTAGGGACCCTAGTTCTTA
				UR	GGTTTAAXTTACTCTACCAGTGGGCGAAGGATTATCTGAAC
	CYP88H2v2	CYP88	85 clan	F	CAAAGAGCAAACAATGGGGTTGG
				R	CTACTTCAATTTTGTGATTTTGACCAGATAA
				UF	GGCTTAAXATGGAATTGACCAACGTCATAGGAT
				UR	GGTTTAAXCTACTTCAATTTTGTGATTTTGACCAGATAA
	CYP88H12	CYP88	85 clan	F	ATGGAGTTGTTGAGAGTGTTAGGATG
				R	CAATATCGGTTATTTTGGGAGTTTAGTGA
				UF	GGCTTAAXATGGAGTTGTTGAGAGTGTTAGGATGGG
				UR	GGTTTAAXTTATTTTGGGAGCTTAGTGATTTTCACGAGATA
	CYP88A89	CYP88	85 clan	F	ATGGAATTGACCAACGTCATAGGAT
				R	GATGAACATTTTGTGATCCTTGCGAC
				UF	GGCTTAAXATGGGGTTGGGGTGGATGGTT
				UR	GGTTTAAXTTACGCAGATGATGAACATTTTGTGATCCTT
	CYP88H7	CYP88	85 clan	F	ATGGAGTACTCAAGCATGTTAGGATT
				R	CACTCGAGAGCTTGATGATTTTGG
				UF	GGCTTAAXATGTTAGGATTGGCTAGCACTCTCA
				UR	GGTTTAAXCTAGAGATCACTCGAGAGCTTGATGATT

(Table S1 continued)

Type	Gene	Family	Clan	Primer type	Sequence
	CYP88H8	CYP88	85 clan	F	CGCAATTAAGACTAGAGCGAGAGAGAAATAC
				R	CCACACGGCTTAATTATTATGATGACACT
				UF	GGCTTAAXATGGAGTTATTCAACAACAAGCTAGATTT
				UR	GGTTTAAXTTATGATGACACTTTGGTGAGTTTCACTA
	CYP88H11	CYP88	85 clan	F	ATGGAGTTGTTGAGAGTGTTAGGATG
				R	CAATATCGGTTATTTTGGGAGTTTAGTGA
				UF	GGCTTAAXATGGAGTTGTTGAGAGTGTTAGGATGGG
				UR	GGTTTAAXTTATTTTGGGAGCTTAGTGATTTTCACGAGATA
	CYP88A88	CYP88	85 clan	UF	GGCTTAAXATGGAGTTAGGGTCTGTGCTTATATGCA
				UR	GGTTTAAXTCATGACGAACATTTATTGATCCTCGCC
	CYP88A44v1	CYP88	85 clan	UF	GGCTTAAXATGGAGTTAGGGTCTGTGCTTATATGCA
				UR	GGTTTAAXTCATGACGAACATTTATTGATCCTCGCC
	CYP88A44v2	CYP88	85 clan	UF	GGCTTAAXATGGAGTTAGGGTCTGTGCTTATATGCA
				UR	GGTTTAAXTCATGACGAACATTTATTGATCCTCGCC
leaf	CYP88H6	CYP88	85 clan	UF	GGCTTAAXATGGAGTATTCAAGCGTGTTAGGATTGG
				UR	GGTTTAAXTTAGAGCTTGATGATTTTGGCAAGAAAGTTGTC
leaf	CYP88H1	CYP88	85 clan	UF	GGCTTAAXATGGAGTTATTCAACGACAAGCTAGATT
				UR	GGTTTAAXTTATGATGATGATACTTTTGTGAGTTTCACCAA
leaf	CYP88H10	CYP88	85 clan	UF	GGCTTAAXATGGAGTTGTTGAGAGTGTTAGGATGGG
				UR	GGTTTAAXTTATTTTGGGAGCTTAGTGATTTTCACGAGATA
leaf	CYP88A43	CYP88	85 clan	UF	GGCTTAAXATGGAGTTAGGGTCTGTGCTTATATGCA
				UR	GGTTTAAXTTATGACGAACATTTCTTGATCCTCGCCAAGCA
	CYP88H9	CYP88	85 clan	F	GATGGCAGATATCTTAGAATTGGTC
				R	AGTCCCAAGTGGAGCACTTT
				UF	GGCTTAAXATGGCAGATATCTTAGAATTGGTCCTTG
				UR	GGTTTAAXTTATTTTGGGAGTTTTGTGATTTTGACGAGATA
	CYP716C52	CYP716	85 clan	F	GTTGTGACAAGAATGGGAAAG
				R	AGATTAATTGGCTGAGTGAAGG
				UF	GGCTTAAXATGGGAAAGTACTCCAATGAAGTGTTCA
				UR	GGTTTAAXTTAATTGGCTGAGTGAAGGCGGATTG
	CYP716E53	CYP716	85 clan	F	CTCGTCATTCTCTTAGCCAT
				R	CTTAATTGTCATGAGGCTGT
				UF	GGCTTAAXATGGATCTCCTGTCTCCATACCTTTTTC
				UR	GGTTTAAXTTAATTGTCATGAGGCTGTAAACGAATTGGAAG
	CYP86B39	CYP86	86 clan	F	ACACCCATAACATGCTCTAC
				R	GATGAAAGACCATAGTTATTAAAACA
				UF	GGCTTAAXATGCTCTACAACAACACCACCATGATCA
				UR	GGTTTAAXTTAATAGTTCATCTTAAGGTACTTATGAAGCTC
	CYP96A145	CYP96	86 clan	F	CTTAAGTCATGGCCTTATTAGGTC
				R	GAAGGATGATAAGGGACAGCTA
				UF	GGCTTAAXATGGCCTTATTAGGTCTTCTTGAAATCC
				UR	GGTTTAAXTTAGGCCCATCTCTTACTGACCTTCACCTTCAA

(Table S1 continued)

Type	Gene	Family	Clan	Primer type	Sequence
**Others**	P_{GAL1}			UF	CACGCGAXAGTACGGATTAGAAGC
				UR	ACCCGTTGAXGGGTTTTTTCTCCT
	UBC7 pUG-F			F	GAGATTATCCTAAAAGGAACTTCCCTAGTAATAGTGTAATTTGGAAGGGCATA GCCAGCTGAAGCTTCGTACGC
	pUG-R			R	GTATATAGAGAACAGTTAAAAGGAAGACCAAATGATCATTAACCTGCTACCT GCTTTCAGCATAGGCCACTAGTGGATCTG
	UBC7 prom			F	GAAGAACTTACCAGACTGTTTCAAGT

Table S2. Yeast expression constructs

name	backbone	promoter*	insert	source
pESC TRP EV	pESC TRP	P_{GAL1}		Agilent Technologies, Cat. #217453
pESC TRP CYP712K1	pESC TRP	P_{GAL1}	*TwCYP712K1*	this study
pESC TRP CYP712K2	pESC TRP	P_{GAL1}	*TwCYP712K2*	this study
pESC TRP CYP712K3	pESC TRP	P_{GAL1}	*TwCYP712K3*	this study
pESC LEU EV	pESC LEU	P_{GAL1}		Agilent Technologies, Cat. #217452
pESC LEU TwCPR1	pESC LEU	P_{GAL1}	*TwCPR1*	this study
pUUS EV	pUUS			Jensen et al., 2014[75, a]
pUUS KdFRS	pUUS	P_{GAL1}	P_{GAL1}, *KdFRS*	this study
pHTDH EV	pHTDH	P_{TDH3}		Trikka et al., 2015[50]
pHTD-tHMGR2	pHTDH	P_{TDH3}	*tHMG2*	Trikka et al., 2015[50]

*Promoter included in backbones except for pUUS.
[a]Citation numbers correspond to references in the main article.

Table S3. Assigned ^{1}C and ^{13}C NMR peaks for compound 5 and 6

**Position**	**Compound 5**		**Compound 6**	
	**^{1}H NMR**	**^{13}C NMR**	**^{1}H NMR**	**^{13}C NMR**
	δ_H (J in Hz)	δ_C	δ_H (J in Hz)	δ_C
1	1.66 (m, H-β)	22,4	1.64 (m, H-β)	22,4
	1.94 (m, H-α)		1.92 (m, H-α)	
2	2.28 (m, H-α)	41,4	2.24 (m, H-α)	41,5
	2.36 (m, H-β)		2.31 (m, H-β)	
3		213,3		214,4
4	2.22 (q, J = 6.5)	58,4	2.2 (q, J = 6.5)	58,2
5		42,3		42,2
6	1.27 (m, H-β)	41,7	1.23 (m, H-β)	41,2
	1.72 (m, H-α)		1.68 (m, H-α)	
7	1.38 (m, H-β)	18,4	1.28 (m, H-β)	18,2
	1.46 (m, H-α)		1.43 (m, H-α)	
8	1.38 (m)	53,5	1.33 (m)	50,6
9		37,6		37,5
10	1.51 (m)	59,6	1.49 (m)	59,5
11	1.25 (m, H-β)	35,8	1.25 (m, H-β)	35,2
	1.45 (m, H-α)		1.40 (m, H-α)	
12	1.13 (m, H-β)	29,9	1.16 (m, H-β)	30,4
	1.45 (m, H-α)		1.48 (m, H-α)	
13		39,6		39,3
14		38,4		39,3
15	1.26 (m, H-β)	32,9	1.28 (m, H-β)	29,8
	1.51 (m, H-α)		1.43 (m, H-α)	
16	1.30 (m, H-β)	36	1.34 (m, H-β)	36,2
	1.51 (m, H-α)		1.61 (m, H-α)	
17		29,9		30,1
18	1.59 (m)	42	1.5 (m)	44,3
19	1.34 (m)	30,7	1.45 (m)	29,5
20		33,2		40,4
21	1.34 (m)	27,9	1.28 (m)	29,4
22	0.97 (m, H-β)	39,6	0.91 (m, H-β)	36,8
	1.40 (m, H-α)		1.92 (m, H-α)	
23	0.85 (d, J = 6.5 Hz)	6,9	0.80 (d, J = 6.5 Hz)	6,7
24	0.70 (s)	14,8	0.65 (s)	14,6
25	0.86 (s)	18	0.93 (s)	17,7
26	1.03 (s)	18,6	0.81 (s)	18,4
27	1.00 (s)	20,9	0.81 (s)	16,4
28	1.20 (s)	32,2	1.03 (s)	31,8
29	3.24 (sl)	74,9		182,3
30	1.01 (s)	25,9	1.15 (s)	31,6

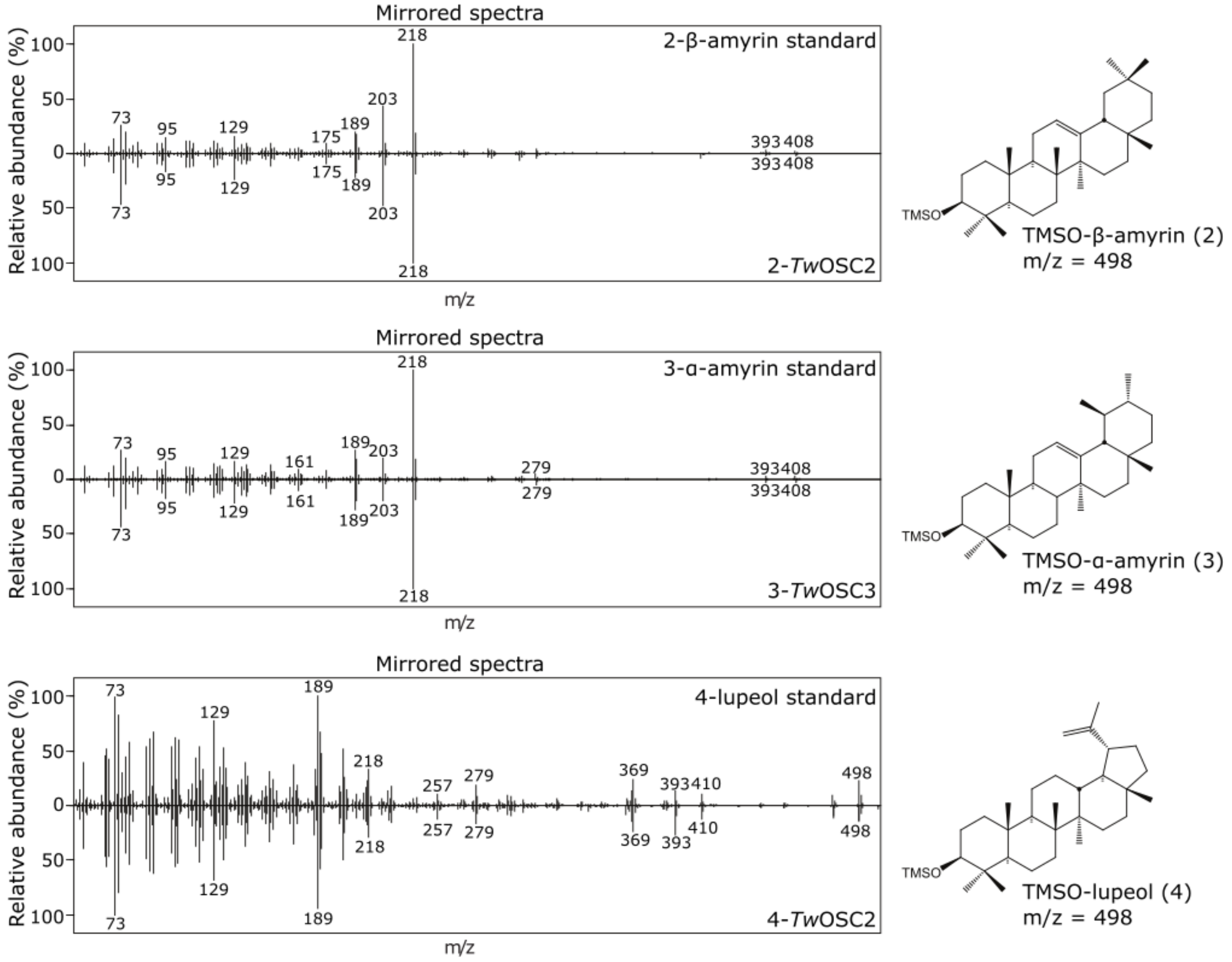

Figure S1. Comparison of the mass spectra of the TMS derivatized products of the *Tw*OSC in tobacco leaf infiltration experiments with the spectra of TMS derivatized authentic standards.

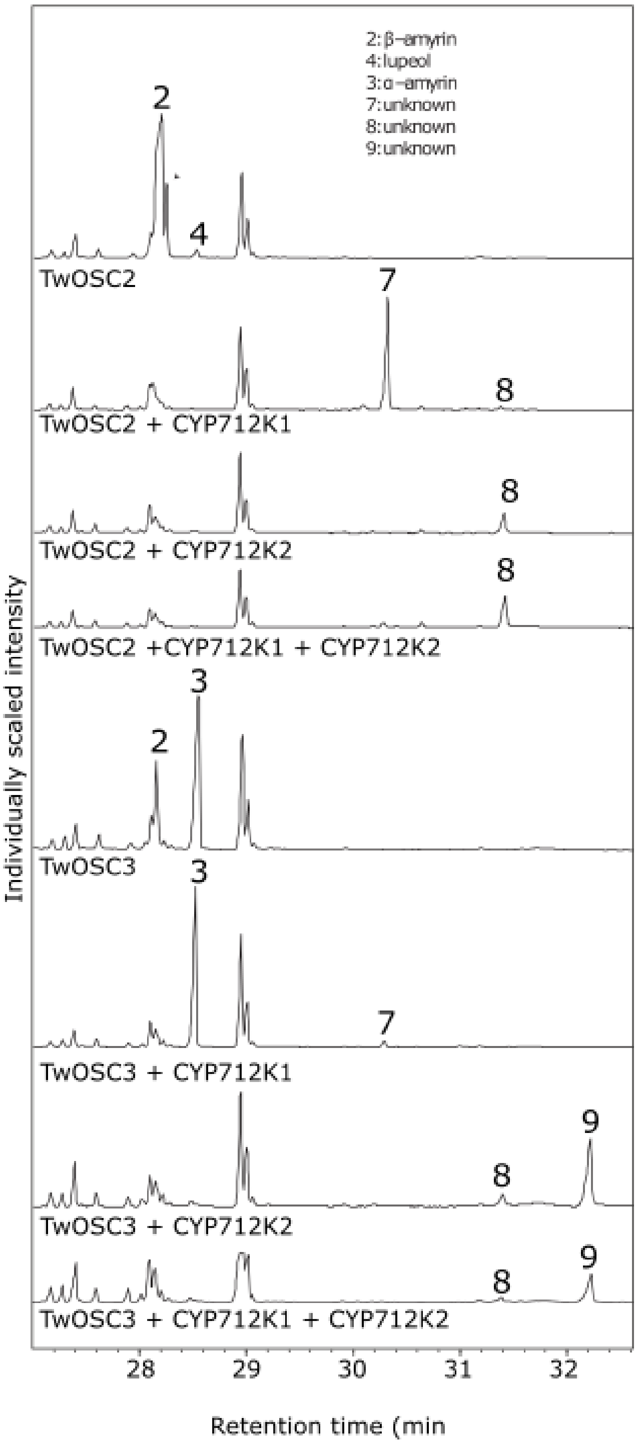

Figure S2. Transient expression of *T. wilfordii* CYP712K1 and CYP712K2 to monitor their ability to oxygenate the core structures produced by *Tw*OSC2 and *Tw*OSC3. Two leaf discs (Ø3 cm) of leaves co-expressing the genes encoding the indicated enzymes were extracted in 1 mL ethyl acetate. A 0.15 mL aliquot of this extract was dried, TMS derivatized and analyzed by GC-MS. None of the novel peaks **7-9** had mass spectra or retention times matching any of the analyzed authentic standards (betulinic acid, hederagenin, or oleanolic acid; not shown).

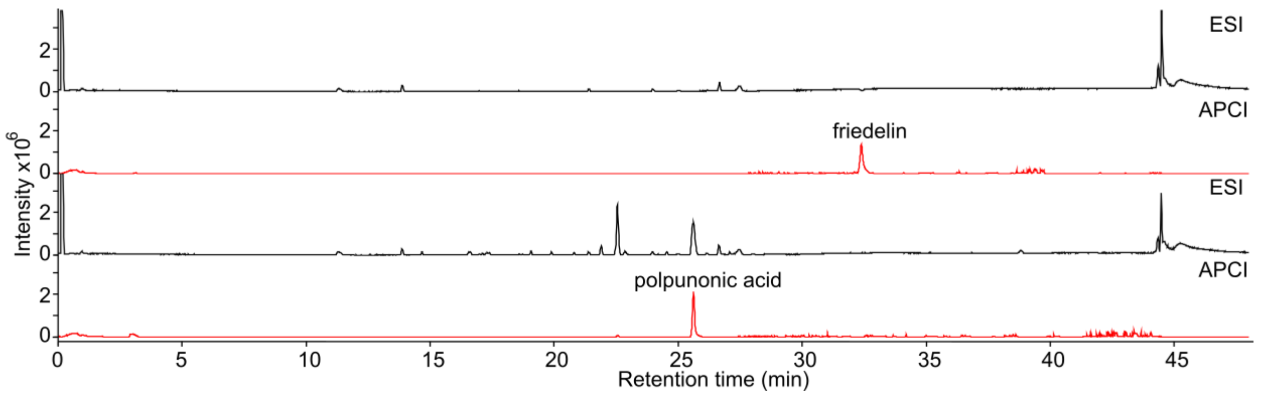

Figure S3. Comparison of LC-ESI-MS and LC-APCI-MS (base peak chromatogram) for the reliable quantification of friedelin and polpunonic acid standards. The same standards (30 µg/mL of either friedelin or polpunonic acid) were analyzed using the same LC-MS equipment and program with the exception of ionization probe (ESI; black trace and APCI; red trace).
